# Excystment-Dependent Alteration of Protein Expression in Terrestrial Ciliate *Colpoda cucullus*

**DOI:** 10.1264/jsme2.ME12200

**Published:** 2013-04-26

**Authors:** Yoichiro Sogame, Katsuhiko Kojima, Toshikazu Takeshita, Eiji Kinoshita, Ryoji Funadani, Tatsuomi Matsuoka

**Affiliations:** 1 Department of Biological Science, Faculty of Science, Kochi University, Kochi 780–8520, Japan; 2 Department of Microbiology and Immunology, Shinshu University School of Medicine, 3–1–1 Asahi, Matsumoto, Nagano 390–8621, Japan; 3 Department of Functional Molecular Science, Graduate School of Biomedical Sciences, Hiroshima University, Kasumi 1–2–3, Hiroshima 734–8553, Japan

**Keywords:** *Colpoda*, excystment, DEAD-box RNA helicase

## Abstract

Protein expression during the excystment of *Colpoda cucullus* was studied by SDS-PAGE. The expression levels of 60-, 50- and 49-kDa proteins were markedly changed from the early to later stage of excystment. The 60-kDa protein (p60) was temporarily expressed first, and its expression was inhibited by actinomycin D. LC-MS/MS analysis showed that the amino acid sequences of p60 partially coincided with those of the *Paramecium tetraurelia* unnamed protein homologous to DEAD-box RNA helicase. These results suggest that p60 expression is enhanced by transcriptional regulation and may be involved in initiating the molecular events leading to cellular morphogenesis.

The mature resting cyst of the terrestrial ciliate *Colpoda cucullus* is characterized by a compact cell structure surrounded by a cyst wall consisting of an outermost layer (*i.e.*, ectocyst) and several inner layers (*i.e.*, endocyst) (8), and is resistant to desiccation, high temperatures, freezing, acid exposure, etc. (10, 11, 13). The central region of the cyst’s cell interior is occupied by numerous electron-lucent reserve material-like granules while the peripheral region contains mitochondria without membrane potential (8). Cytoskeletal elements such as ciliary structures disappear in the resting cyst (8). It has already been found that, in *C. cucullus*, the expression level of a 49-kDa protein (p49; elongation factor 1α [EF-1α] is enhanced, whereas that of 50-kDa protein (p50; mitochondrial ATP synthase β chain) is reduced 2–5 h after the onset of encystment induction (12).

The excystment process in *C. cucullus* involves marked cellular morphogenesis to reconstruct a cilia-bearing vegetative cell structure. The earliest visible event is the dispersal of electron-lucent reserve material-like granules accumulated in the central region and mitochondria aggregated in the peripheral region, which occurs 30 min after the onset of excystment induction (11). The dispersal of organelles by cyclosis is immediately followed by the pulsation of a contractile vacuole (7). At 30–60 min after the onset of excystment induction, the vacuoles containing the electron-lucent granules fuse with the plasma membrane to excrete their contents, and thereafter the endocyst is digested (11). Cellular morphogenesis, including reconstruction of the cilia, is completed within 1.5 h in the most rapid cases (11). Finally, a motile cell emerges from a resting cyst by rupture of the outermost cyst wall (ectocyst) in response to inner pressure from the expansion of the ‘excystment vacuole’ (7). In the present study, we found that the expression level of 60-kDa protein (p60) was elevated in the early stage of excystment of *C. cucullus*. The aim of this work was to identify this protein by liquid chromatography tandem mass spectrometry (LC-MS/MS) analysis and to speculate on its role in excystment.

*Colpoda cucullus* (Nag-1 strain) collected from the soil surface in Kochi Prefecture, Japan was cultured in a 0.05% (w/v) infusion of dried wheat leaves. This wheat-leaf infusion was periodically inoculated with a non-pathogenic strain (6081) of *Klebsiella pneumoniae*, which was cultured on agar plates containing 1.5% (w/v) agar (Wako Pure Chemical Industries, Osaka, Japan), 0.5% (w/v) polypeptone (Nihon Pharmaceutical, Tokyo, Japan), 1% (w/v) beef extract (Becton Dickinson, Franklin Lakes, NJ, USA), and 0.5% (w/v) NaCl. For encystment induction, the cells of *C. cucullus* cultured for 1–2 d were pelleted at 1,500×*g* for 2 min, and resuspended at 50,000 cells mL^−1^ in an encystment-inducing medium containing 1 mM Tris-HCl (pH 7.2) and 0.1 mM CaCl_2_. Cell density was determined by directly counting cells in a given volume of cell suspension under a dissecting microscope. Excystment was induced by replacing the surrounding medium of 1-week-old cysts by a 0.2% (w/v) infusion of wheat leaves.

Actinomycin D (Wako Pure Chemical Industries) was dissolved in dimethyl sulfoxide (DMSO) to give 10,000 μg mL^−1^ stock solution. The stock solution was diluted 1,000 times with excystment-inducing medium (final concentration: 10 μg mL^−1^ actinomycin D containing 0.1% DMSO).

Sodium dodecyl sulfate-polyacrylamide gel electrophoresis (SDS-PAGE) was performed basically according to Laemmli’s method (9). The cysts were disrupted in SDS-PAGE sample buffer (1% [w/v] SDS, 30 mM Tris-HCl, pH 6.8, 5% [v/v] 2-mercaptoethanol and 10% [v/v] glycerol) using an As One Model 226A microhomogenizer (As One, Osaka, Japan) in a microfuge tube on ice, and then boiled for 3 min. A sample containing ~50 μg protein, corresponding to ~5,000 cysts in each lane, was electrophoresed on a 10% gel at 150 V. The gels were stained with 0.2% Coomassie brilliant blue R250 dissolved in a solution containing 45% (v/v) methanol and 10% (v/v) glacial acetic acid, and then de-stained in a 27% (v/v) methanol, 9% (v/v) glacial acetic acid solution.

For two-dimensional polyacrylamide gel electrophoresis (2-D PAGE), the samples disrupted by a sonicator (Bioruptor UCD-200; CosmoBio, Tokyo, Japan) were mixed with an equal volume of double-strength SDS-PAGE sample buffer, boiled for 3 min, and then mixed with an IEF (isoelectric focusing) sample buffer (8 M urea, 1.9% [v/v] Triton X-100 [Wako Pure Chemical Industries], 2% [v/v] Pharmalyte [GE Healthcare, Little Chalfont, UK] and 5% [v/v] 2-mercaptoethanol) at the ratio of 1:5. The IEF disc gel contained 8 M urea, 4.5% (w/v) acrylamide, 0.2% (w/v) N,N′-methylenebisacrylamide, 0.01% (v/v) TEMED (Wako Pure Chemical Industries), 1.9% (v/v) Triton X-100 (Wako Pure Chemical Industries), 4.7% (v/v) Pharmalyte, and 0.07% (w/v) ammonium persulfate. A sample containing proteins (~10 mg) corresponding to about 1,000,000 cysts was analyzed in IEF at 100 V for 2 h and subsequently at 400 V for 4 h at room temperature. After IEF, the gel was soaked twice for 30 min each in double-strength SDS-PAGE sample buffer, and then electrophoresed on a 10% gel.

The proteins separated on polyacrylamide gels were transferred for 60 min at 100 mA onto an Immobilon-P transfer membrane (EMD Millipore, Billerica, MA, USA) using a Hoefer TE70 semi-dry blotting system (GE Healthcare) with three kinds of blotting solutions: solution A, 300 mM Tris containing 20% (v/v) methanol; solution B, 25 mM Tris containing 20% (v/v) methanol; and solution C, 25 mM Tris-borate buffer, pH 9.5, containing 20% (v/v) methanol. Blots were stained with a stain containing 0.1% (w/v) Coomassie brilliant blue R250, 40% (v/v) methanol and 1% (v/v) glacial acetic acid, followed by de-staining in a 50% (v/v) methanol solution.

Prior to LC-MS/MS analysis, the proteins separated by 2D-PAGE were electroblotted onto an Immobilon-P transfer membrane. Protein spots visualized by CBB staining were cut out and then subjected to reduction with 40 mM DTT (dithiothreitol) for 1 h at 37°C and alkylation with 100 mM iodoacetic acid for 20 min at room temperature to generate carboxymethylation of cysteine residues. To block non-specific binding of protease, membrane pieces were treated with 0.5% PVP40 (polyvinylpyrrolidone) in 100 mM acetic acid for 30 min at 37°C. On-membrane digestion of proteins was performed in 10 μL of 30 mM Tris-HCl (pH 8.5) containing 10% acetonitrile with 1 pmol trypsin (Sigma-Aldrich, St. Louis, MO, USA) for 18 h at 37°C. Peptides produced by protease digestion were separated by a 0–40% linear gradient with acetonitrile for 60 min and analyzed with a Waters UPLC Xevo Qtof. Raw data were processed with Waters ProteinLynx Global Server 2.4. Subsequently, protein was identified by searching against the Alveolata protein sequences registered in NCBI Entrez protein records using PEAKS online 5.3 (Bioinformatics Solutions, Waterloo, ON, Canada).

Fig. 1A shows changes in the total protein composition contained in excystment-induced *C. cucullus* cells, as analyzed by SDS-PAGE. In this case, the vegetative cells began to emerge within 60 min, and most cells emerged within 300 min of the onset of excystment induction. The most marked and earliest event was the temporary enhancement of the expression of a 60-kDa protein (p60) 10 min after the onset of excystment induction (Fig. 1A). This protein, which was expressed first in the excystment process, may play a role in initiating the molecular events leading to morphogenetic transformation into a vegetative cell. The expression of this p60 was clearly suppressed 15 min after the onset of excystment induction in the presence of 10 μg mL^−1^ actinomycin D (Fig. 1B). This result suggests that the excystment-specific expression of p60 occurs by transcriptional regulation. In the later stage (60–180 min after the onset of excystment induction), alterations in the expressions of 50-kDa and 49-kDa proteins (p50 and p49, respectively) were observed; p50, which had disappeared during encystment, was expressed again, while the expression of p49, which had been enhanced during encystment (12), was reduced. The alteration of the expression levels of these proteins during encystment is suggested to be regulated transcriptionally (12). Previous LC-MS/MS analysis revealed that p50 is a mitochondrial ATP synthase β chain, and that p49 is elongation factor 1α (EF-1 α) (12). EF-1 α is reported to be multifunctional, such as actin or microtubule bundling activity, the regulation of proteasome-dependent degradation of proteins, longevity, oxidative stress-induced apoptosis, cold acclimation, etc. (4). At least *Colpoda* EF-1 α (p49) may be indispensable for the formation and/or maintenance of the resting cyst, although the specific function of this protein in the encystment process is still unknown. In *C. cucullus*, mitochondrial membrane potential, which had disappeared during encystment (8), was regained during excystment (data not shown). In *Trypanosoma*, based on the result that knock-down of the mitochondrial ATP synthase α or β chain reduces mitochondrial membrane potential, the ATP synthase is suggested to be involved in the maintenance of mitochondrial membrane potential (1). The expression of the *C. cucullus* mitochondrial ATP synthase β chain (p50) during excystment may be responsible for recovery of the mitochondrial membrane potential.

A sample (Fig. 1C-1) in which excystment had not yet been induced and another sample (Fig. 1C-2) obtained from cysts 15 min after the onset of excystment induction were analyzed on 2-D PAGE. The expression of p60 was enhanced by excystment induction. The spot corresponding to p60 on the transfer membrane (Fig. 1C-2) was analyzed by LC-MS/MS, followed by a database search. In the case of p60, the amino acid sequences of one of the protease-digested fragments completely coincided with, and three fragments partially coincided with the sequences of the *Paramecium tetraurelia* unnamed protein (Table 1), whose sequence was homologous to DEAD-box RNA helicase.

The RNA helicases of the multifunctional DEAD-box protein family are known to participate in events involving RNA, such as transcriptional regulation, by interacting with transcription factors and nuclear receptors, degradation of RNA, etc. (3). In the RNA degradation process, the DEAD-box proteins were shown to be involved in the generation of certain microRNAs (miRNAs) (6), which modulate gene expression by partially base-pairing with target mRNA to degrade them (2, 5). The *Colpoda* p60 may be involved in the transcriptional regulation of the genes that act in excystment events, and/or in the degradation of resting cyst-specific mRNA. At least, the finding that *Colpoda* p60 is the protein expressed first in the excystment process implies that this protein may play an important role in initiating the molecular events leading to cellular morphogenesis.

## Figures and Tables

**Fig. 1:**
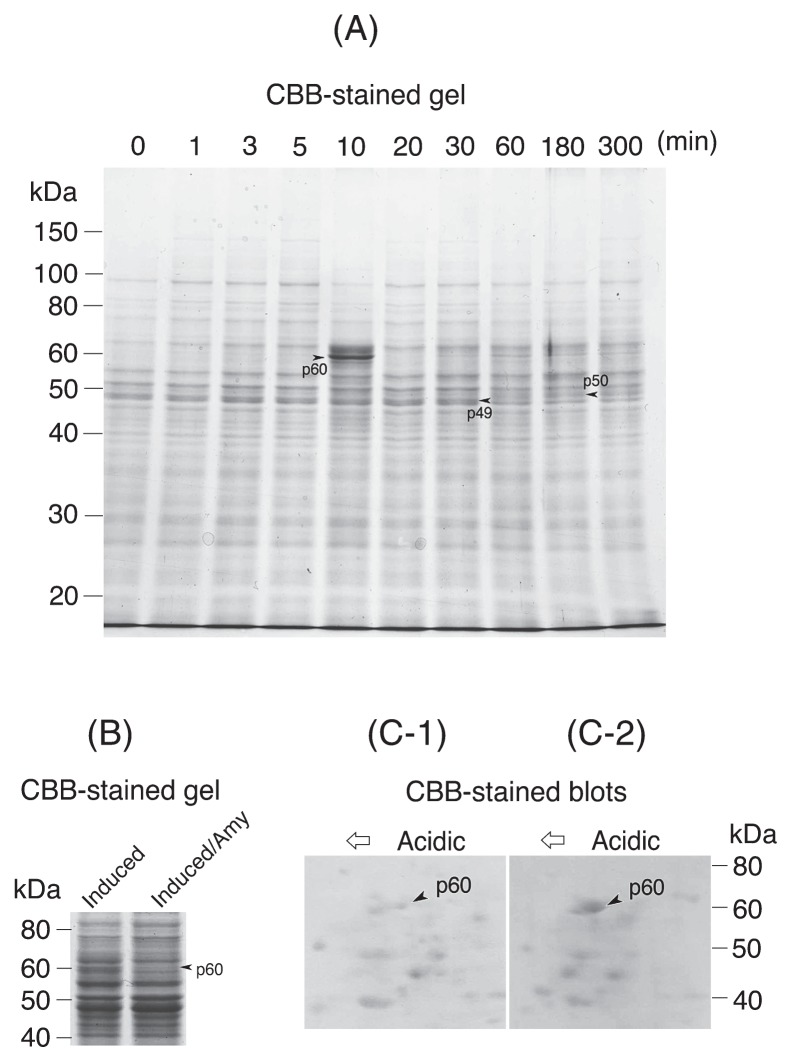
Protein expression in excystment-induced *Colpoda cucullus*. (A) SDS-PAGE (gel) of total proteins contained in excysting cells. Cysts were solubilized with a sample buffer at 0 to 300 min (labeled at the top of each lane) after the onset of excystment induction. (B) SDS-PAGE (gel) of total proteins of excystment-induced cysts showing the effects of actinomycin D on the expression of proteins. ‘Induced’, excysting cells 15 min after the onset of excystment induction; ‘Induced/Amy’, excysting cells 15 min after the onset of excystment induction in the presence of 10 μg mL^−1^ actinomycin D. (C) 2-D PAGE (blots) of the proteins contained in a sample without excystment induction (C-1) and 15 min after the onset of excystment induction (C-2), respectively.

**Table 1:** *Colpoda cucullus* p60, identified by LC-MS/MS, whose expression is modified by excystment induction

Protein name	Sequence of exactly matched peptides	*Partially matched peptides	Accession no. (organism)	Sequence coverage (%)
Unnamed protein (homologous to DEAD-box proteins)	LQDADYDK	LNLEPIIQKVIANEFKFAIGGTSNE TDRDIIEKGK	gi 124409163 (*Paramecium tetraurelia*)	7 (43AA/579AA)

* *De novo* sequences. The residues matching those in sequences predicted by PEAKS online 5.3 are underlined.

## References

[1] Brown SV, Hosking P, Li J, Williams N (2006). ATP synthase is responsible for maintaining mitochondrial membrane potential in bloodstream form *Trypanosoma brucei*. Eukaryot Cell.

[2] Carthew RW (2006). Gene regulation by microRNAs. Curr Opin Genet Dev.

[3] Cordin O, Banroques J, Tanner NK, Linder P (2006). The DEAD-box protein family of RNA helicases. Gene.

[4] Ejiri S (2002). Moonlighting functions of polypeptide elongation factor 1: From actin bundling to zinc finger protein R1-associated nuclear localization. Biosci Biotechnol Biochem.

[5] Filipowicz W, Jaskiewicz L, Kolb FA, Pillai RS (2005). Post-transcriptional gene silencing by siRNAs and miRNAs. Curr Opin Struct Biol.

[6] Fukuda T, Yamagata K, Fujiyama S (2007). DEAD-box RNA helicase subunits of the Drosha complex are required for processing of rRNA and a subset of microRNAs. Nature Cell Biol.

[7] Funadani R, Suetomo Y, Matsuoka T (2012). Emergence of the terrestrial ciliate *Colpoda cucullus* from a resting cyst: Rupture of the cyst wall by active expansion of an excystment vacuole. Microbes Environ.

[8] Funatani R, Kida A, Watoh T, Matsuoka T (2010). Morphological events during resting cyst formation (encystment) in the ciliated protozoan *Colpoda cucullus*. Protistology.

[9] Laemmli U (1970). Cleavage of structural proteins during the assembly of the head of bacteriophage T4. Nature.

[10] Maeda H, Akematsu T, Fukui R, Matsuoka T (2005). Studies on the resting cyst of ciliated protozoan *Colpoda cucullus*: Resistance to temperature and additional inducing factors for en-or excystment. J Protozool Res.

[11] Sogame Y, Kida A, Matsuoka T (2011). Possible involvement of endocyst in tolerance of the resting cyst of *Colpoda cucullus* against HCl. Afr J Microbiol Res.

[12] Sogame Y, Kojima K, Takeshita T, Kinoshita E, Matsuoka T (2012). EF-1α and mitochondrial ATP synthase β chain: Alteration of their expression in encystment-induced *Colpoda cucullus*. J Euk Microbiol.

[13] Taylor CV, Strickland AGR (1936). Effects of high vacua and extreme temperatures on the cysts of *Colpoda cucullus*. Physiol Zool.

